# Enhanced intraparietal phase-lag synchronization in the high-gamma band during retention of visuospatial working memory

**DOI:** 10.1007/s13534-025-00547-8

**Published:** 2026-01-17

**Authors:** Jimin Park, Sangjun Lee, Seonghun Park, Chany Lee

**Affiliations:** 1 Department of Psychiatry, University of North Carolina, Chapel Hill, NC USA; 2 Department of Biomedical Engineering, University of Minnesota, Minneapolis, MN USA; 3 Experimental Psychology Lab, Department of Psychology, Carl von Ossietzky University, Oldenburg, Germany; 4 Cognitive Science Research Group, Korea Brain Research Institute, 61 Cheomdan-ro, Dong-gu, 41068 Daegu, Republic of Korea

**Keywords:** Visuospatial working memory (VWM), Electroencephalography (EEG), Weighted phase lag index (wPLI), Transcranial alternating current stimulation (tACS), Multi-site multi-phase transcranial alternating current stimulation (msmp-tACS), Brain lateralization

## Abstract

Although zero-phase lag between cortical regions has been generally regarded as the optimal state, it has also been suggested that a non-zero phase delay of electroencephalography (EEG) signals in the gamma frequency band between bilateral parietal areas may have a significant meaning. Indeed, the phase delays of the gamma band between the cortical regions are reportedly associated with the direction of communication between the regions. In this study, we aimed to demonstrate synchrony with phase lag between cortical regions involved in visuospatial working memory (VWM) performance. We used EEG to compute the weighted phase lag index (wPLI) from the EEG signals concurrently recorded during the VWM task. An increase in wPLI value between the electrodes positioned over the bilateral parietal areas was observed during the VWM task. The wPLI values positively correlated with the lateralization index (LI) between the left and right visual hemifields. Furthermore, event-related desynchronization of gamma band activity is observed when wPLI peaked. Our findings suggest that phase lagged synchronization of high gamma band over bilateral parietal areas may reflect which information to prioritize during processing of VWM.

## Introduction

Working memory (WM) is a complex cognitive function that enables temporary retention and manipulation of information. The Visuospatial component of working memory (VWM) specifically supports storage and comparisons of visual information to guide goal directed behavior (i.e., visual search [1]). Such a function of VWM is critical for stable visual perception because VWM is required for the preservation of information during blinks and saccades [2], and is also known to show positive correlation with other higher order cognitions such as fluid intelligence [3, 4].

Neuroimaging studies consistently implicate that that the core brain regions responsible for VWM are the occipital, frontal, and parietal regions [5–7]. Especially, the frontal and parietal regions, or the fronto-parietal network, are reported to be associated with goal-directed control of VWM. Parietal areas (posterior parietal cortex [PPC] and intraparietal sulcus [IPS]) and frontal areas (dorsolateral prefrontal cortex [DLPFC]), are associated with the temporary storage of information and manipulation of information, respectively [8, 9]. Specifically, parietal regions play a central role in visual information storage and capacity control, whereas the frontal regions are more responsible for integration and processing of information for decision making.

Meanwhile, transcranial electrical stimulation (tES) studies have demonstrated significant modulation of WM capacity by targeting the parietal areas [8–12]. When transcranial alternating current stimulation (tACS) was applied, their effects on WM capacity were highly dependent on the properties of the injection currents. For instance, 4-Hz and 7-Hz tACS showed different after-effects on the WM capacity [10]. Additionally, phase-specific effects of tACS on WM have also been reported for multi-site tACS [8]. TACS entrains endogenous brain oscillations to the injected current in frequency- and phase- specific manners [13], and therefore selection of appropriate stimulation parameters based on the electrophysiological evidences are critical in the successful modulation of target brain functions.

Several tACS studies attempted to simultaneously stimulate two cortical areas using alternating currents (ACs) [8, 14–16]. A multi-site, multi-phase tACS (msmp-tACS) which targeted phase delays at high gamma (80 Hz) over bilateral parietal cortex showed change in working memory strategy by lateralizing the performance towards visual hemifield contralateral to the stimulation site with phase lead [17]. However, the study did not report any electrophysiological evidence that shows connection between the proposed behavioral change and activity between the bilateral parietal cortex.

In this study, we aimed to investigate the relationship between the gamma oscillation of bilateral parietal cortex and the lateralization of visual information during visuospatial working memory task. Because the previous tACS studies showed correlation between behavioral lateralization and induced phase delay over the parietal areas, we first calculated the weighted phase lag index (wPLI) from electroencephalography (EEG) signals recorded during a VWM task performance. The wPLI is a functional connectivity measure that entails consistency of phase differences between two sites of interest. We subsequently investigated whether the functional connectivity observed over the bilateral parietal areas correlated with the VWM performance. Finally, we calculated gamma power during the task to observe event related synchronization and desynchronization (ERS and ERD, respectively) of the gamma rhythms.

## Methods

### Participants

Twenty-one healthy volunteers participated in the EEG experiment (5 women and 16 men, age: 25.03 ± 2.55). Number of participants were decided based on previous study reporting correlation of EEG to working memory [18], with assumption of two-tailed test, type-I error of 0.05, and power of 0.8. Individuals with any identifiable neurological disorder, head injury, or any personal or family history of psychiatric illness were excluded from participating in the study. Participants signed a consent form before participation. The study protocol was approved by the institutional review board of Hanyang University, South Korea (IRB No. HYU-2020-010). All the participants received monetary compensation for their participation in the experiments. Two participants were excluded from further analyses due to technical issue in data recording and low performance on the VWM task below the chance level, respectively.

### Behavioral task

**Fig. 1:**
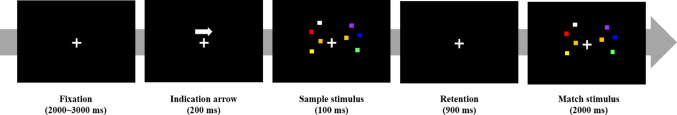
Match to delayed sample task for visuospatial working memory. Fixation of 2000–3000 ms is followed briefly by the indication arrow (200 ms), which informs participants of the hemifield that they should memorize. After the indication arrow disappears, sample stimuli, which is an array of squares, are presented for 100 ms on both hemifields, separated by the fixation cross. A retention period follows, lasting for 900 ms, match stimuli are subsequently presented for 2000 ms. During the presentation of the match stimulus, participants were asked to indicate whether the colors of the squares on the hemifield indicated by the indication arrow matched the colors of sample stimulus

A visual delayed match-to-sample task was employed [3], in which each trial consisted of fixation (2000–3000 ms), indication arrow (200 ms), sample stimulus (100 ms), retention (900 ms), and response stimulus (2000 ms), with the duration of each stage in parentheses (see Fig. 1). Throughout the task, a fixation cross was displayed at the center of the screen. After the initial fixation, an arrow pointing either left or right appeared for 200 ms above the fixation cross. Once the indication arrow disappeared, two sets of square arrays were displayed on the left and right sides of the fixation cross for 100 ms. All the squares in each array had different colors, and the number of squares in the two arrays displayed on both sides of the fixation cross were the same (4–6). During this period, the participants were instructed to memorize the colors of the square array that had been indicated by the arrow. After the offset of the sample stimulus presentation, a short retention period (900 ms) followed. Another square array, the response stimulus, was subsequently displayed on both sides of the fixation cross for 2000 ms. During this period, the participants were instructed to identify whether the colors in the array of squares located at the side that had been indicated by the arrow were the same as those presented in the sample stimulus period by pressing a button on the response pad.

The total number of trials in one experimental session was 120, which were divided into six blocks containing 20 trials each. The six blocks contained four, five, or six squares (two blocks each) in a square array. The number of squares in an array corresponded to the task load. To minimize the potential learning effect, the six blocks were randomly selected from 18 different blocks (six blocks for each task load) using e-Prime 3.0 (E-Prime Ps{Citation}ychology Software Tools Inc., Pittsburgh, USA). Of the 20 trials in each block, ten were left hemifield trials (indicated by the arrow pointing left), whereas the other ten were right hemifield trials (indicated by the arrow pointing right); each hemifield trial consisted of five match and five mismatch trials.

For analysis, accuracy and reaction time were calculated. Furthermore, the K-value, defined as ‘load × (hit rate – false rate)’, was computed to quantify the VWM capacity. The K-value takes the task load into account, which better represents the WM capacity than the accuracy [3, 10]. Finally, the lateralization index (LI) was calculated to analyze the laterality of the WM performance of the visual hemifields using the following equation:

1 $$\mathrm{LI}=\frac{{\mathrm{K}}_{R}-{\mathrm{K}}_{\mathrm{L}}}{{\mathrm{K}}_{\mathrm{R}}+{\mathrm{K}}_{\mathrm{L}}}$$

K_R_ and K_L_ denote the K-values for the right and left hemifield trials, respectively.

### EEG recordings and preprocessing

The EEG data were recorded at a sampling rate of 2048 Hz from eight scalp electrodes (P5, P1, P2, P6, PO7, O1, O2, and PO8), using the Biosemi ActiveTwo system (Biosemi, Amsterdam, The Netherlands). After the recording, the data of the trials highly contaminated with noise were removed manually following the automatic screening of the time intervals with a maximum absolute signal amplitude greater than 100 μV. The most trials removed were 4 (removed 0.47 trials per participant on average). The signal was filtered from 50–70 Hz and 70–90 Hz to represent low and high gamma activity, respectively.

### Weighted phase lag index (wPLI)

To investigate whether a non-zero phase delay existed between interhemispheric parietal regions, the wPLI was computed for four pairs of electrodes positioned symmetrically (P5–P6, P1–P2, PO7–PO8 and O1–O2). The wPLI is an index ranging between 1 and 0, with 1 indicating that the phase delay between two signals is π/2 and 0 indicating that the phase delay is either 0 or π [19]. The equation for computing wPLI (Φ) is

2 $$ {\Phi } = \frac{{\left| {E\left[ {\Delta \varphi } \right]} \right|}}{{E\left[ {\left| {\Delta \varphi } \right|} \right]}}, $$

where Δφ denotes difference of instantaneous phase between two signals.

We computed the wPLI for the EEG data recorded during the VWM task. For each trial, wPLI was computed for 16 segments of 500-ms window size with an overlap of 400 ms, for a 2 s period spanning from 500 ms prior to the sample stimulus onset to 500 ms following the presentation of the response stimulus. Baseline wPLI values were defined as averages of the wPLI values obtained for each trial in time segments of − 0.5 to 0 s, with a timepoint of 0 representing the timepoint of sample stimulus onset.

### Time–frequency analysis and gamma power

We further analyzed changes in gamma power across the task. A morlet wavelet transform with 7 cycles was applied from − 1.5 to 2.5 s relative to stimulus onset. Spectral power was computed for low (50–70 Hz) and high (70–90 Hz) gamma bands. The average activity from − 1.5 to − 1 s were considered baseline. To observe event related changes, gamma power was normalized by dividing by the baseline activity for each trial and then converted to decibel. Finally, computed gamma powers were averaged across trials for each participant.

### Statistical analysis

Since the Kolmogorov–Smirnov test confirmed testing dataset followed normal distribution, paired one sample t-test was conducted for behavioral data and to test the statistical significance between the wPLI values during the VWM task and the baseline wPLI values. Furthermore, the Pearson correlation between the LI and wPLI values during the task was evaluated for each segment. Finally, for all symmetric electrode pairs, two-way analysis of variance (ANOVA) was carried out for the loads (4–6) and hemifields (left and right) with the wPLI values computed for all segments. Subsequently, one-way ANOVA for each within factor was conducted, and then post-hoc analysis of paired one sample t-test was performed, if necessary. Finally, *p*-values were corrected using the false detection rate (FDR) method if multiple comparison correction was necessary.

## Results

### Task performance

**Fig. 2:**
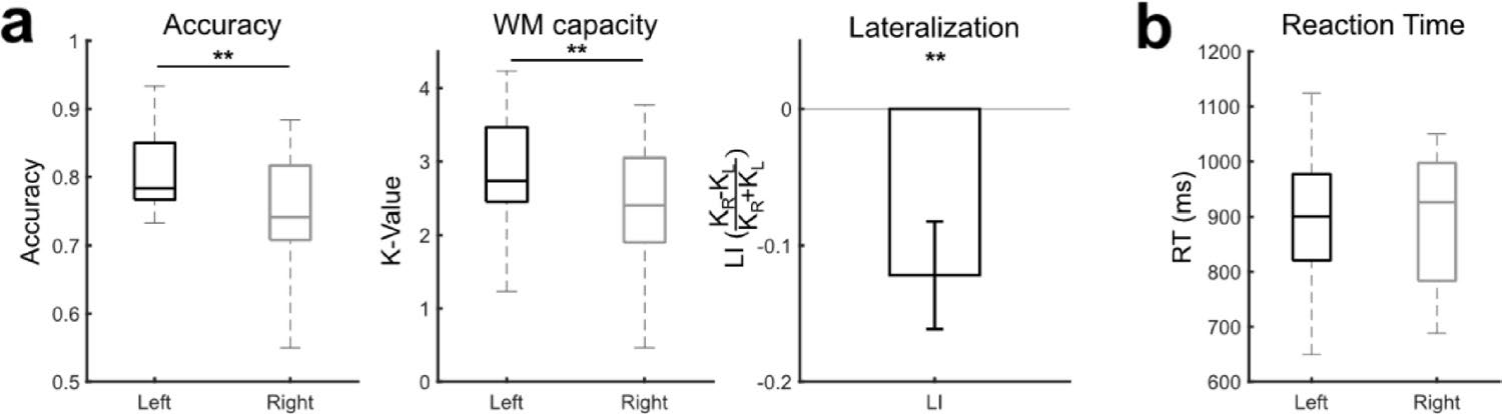
Behavioral performance for the VWM task. **a** accuracy, WM capacity, and computed lateralization, **b** reaction time. ** indicates *p* < 0.01

The accuracy for right and left hemifield trials were 74.69 ± 9.11% and 79.58 ± 6.76%, respectively, showing significant difference between the hemifield (*t*_18_ = − 3.21, *p* < 0.01). Likewise, the working memory capacity was significantly lower for the right hemifield trials compared to left hemifield trials, averaging 0.5 less items memorized (K_R_: 2.38 ± 0.7, K_L_: 2.87 ± 0.91; *t*_18_ = − 3.1, *p* < 0.01). This suggests the participants had tendency to prioritize information from the left visual hemifield compared to right, further evidenced by negative lateralization index (LI: − 0.12 ± 0.16). However, the reaction time for each hemifield was not different (RT_R_: 884.99 ± 126.97 ms, RT_L_: 896.89 ± 122.32 ms; *t*_18_ = − 0.73, *p* = 0.48), which supports that the better performance on the left hemifield trials were due to item storage rather than recall. The behavioral results are depicted in Fig. 2.

### EEG

**Fig. 3:**
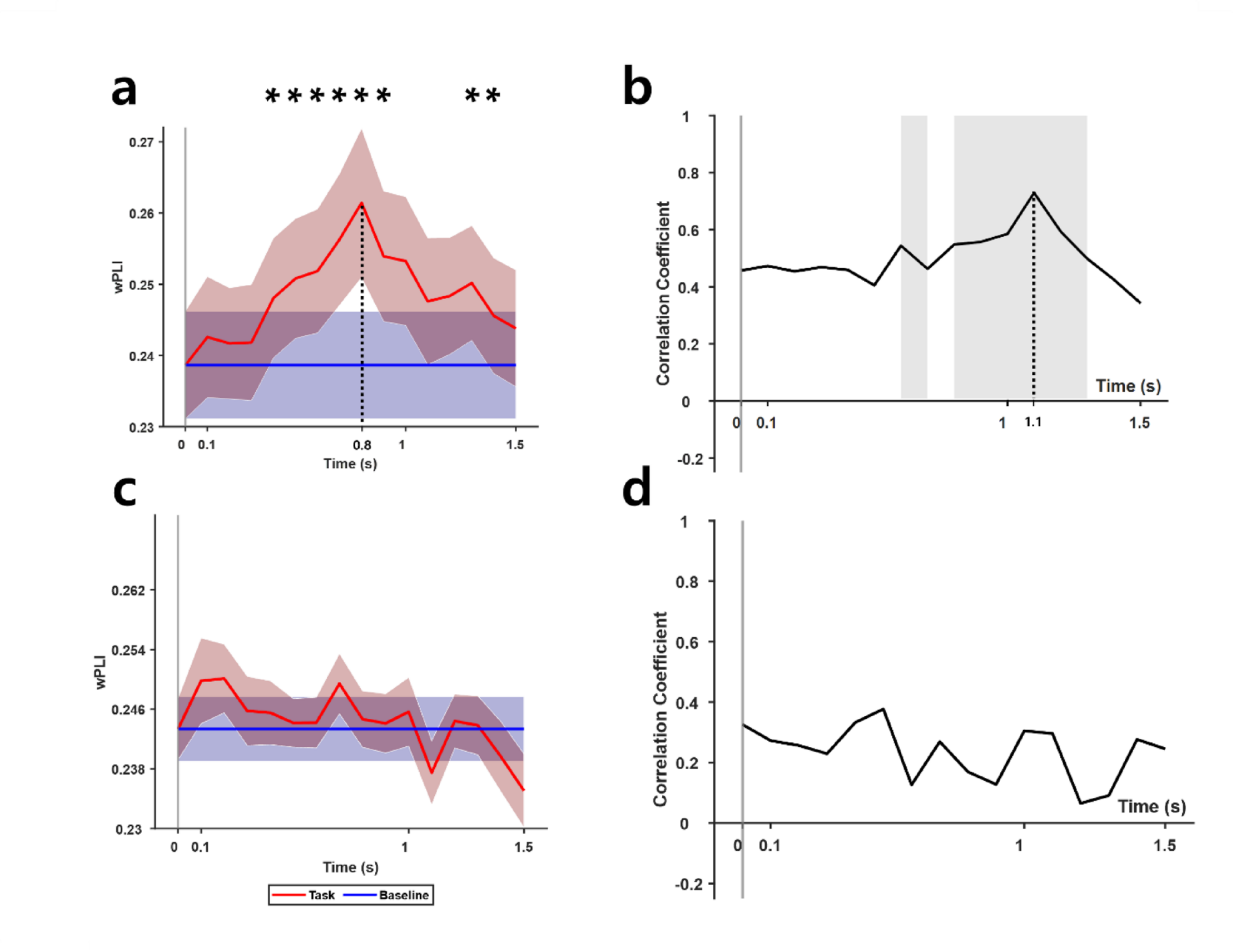
Indices computed by the electroencephalogram recordings from the P1 and P2 electrode pair. The points in the x-axis indicate the end of each time segment, with grey line at time point 0 indicating onset of the sample stimulus. **a**, **b** The mean weighted phase lag index (wPLI) and correlation between the wPLI values of each time window and LI, respectively, computed for high gamma band (70–90 Hz), **c**, **d** The mean weighted phase lag index (wPLI) and correlation between the wPLI values of each time window and LI, respectively, computed for low gamma band (50–70 Hz). Stars denote time windows with wPLI values significantly modulated by task, and shaded regions denote time windows with significant correlation. * indicates *p* < 0.05

At high gamma band, Statistical analysis revealed that the wPLI values for the electrode pairs of P1 and P2 increased significantly for all the segments during the VWM task compared to the baseline (*p* < 0.05). Notably, as depicted in Fig. 3a, the wPLI values were significantly higher during the retention period, peaking at 0.8 s for the P1-P2 electrode pair. Note that the end of each segment is depicted as the time points in Fig. 3. No significant increase in the wPLI during the task was observed for the other electrode pairs. Furthermore, a significant positive correlation between the wPLI values of the P1-P2 electrode pair at high gamma range and LI of the VWM capacity was found for nearly all segments corresponding to retention period, peaking at 1.1 s with a correlation coefficient of 0.72 and decreasing thereafter (Fig. 3b). The time point at which the correlation was maximized corresponded to the end of the retention period. For the low gamma band, no significant task modulation of wPLI was observed, and no significant correlation was found between wPLI values of the P1-P2 electrode pair and LI of the VWM capacity. This shows that the synchrony between intraparietal high gamma activity arose during retention period, when storage and manipulation of memorized items occur.

**Fig. 4:**
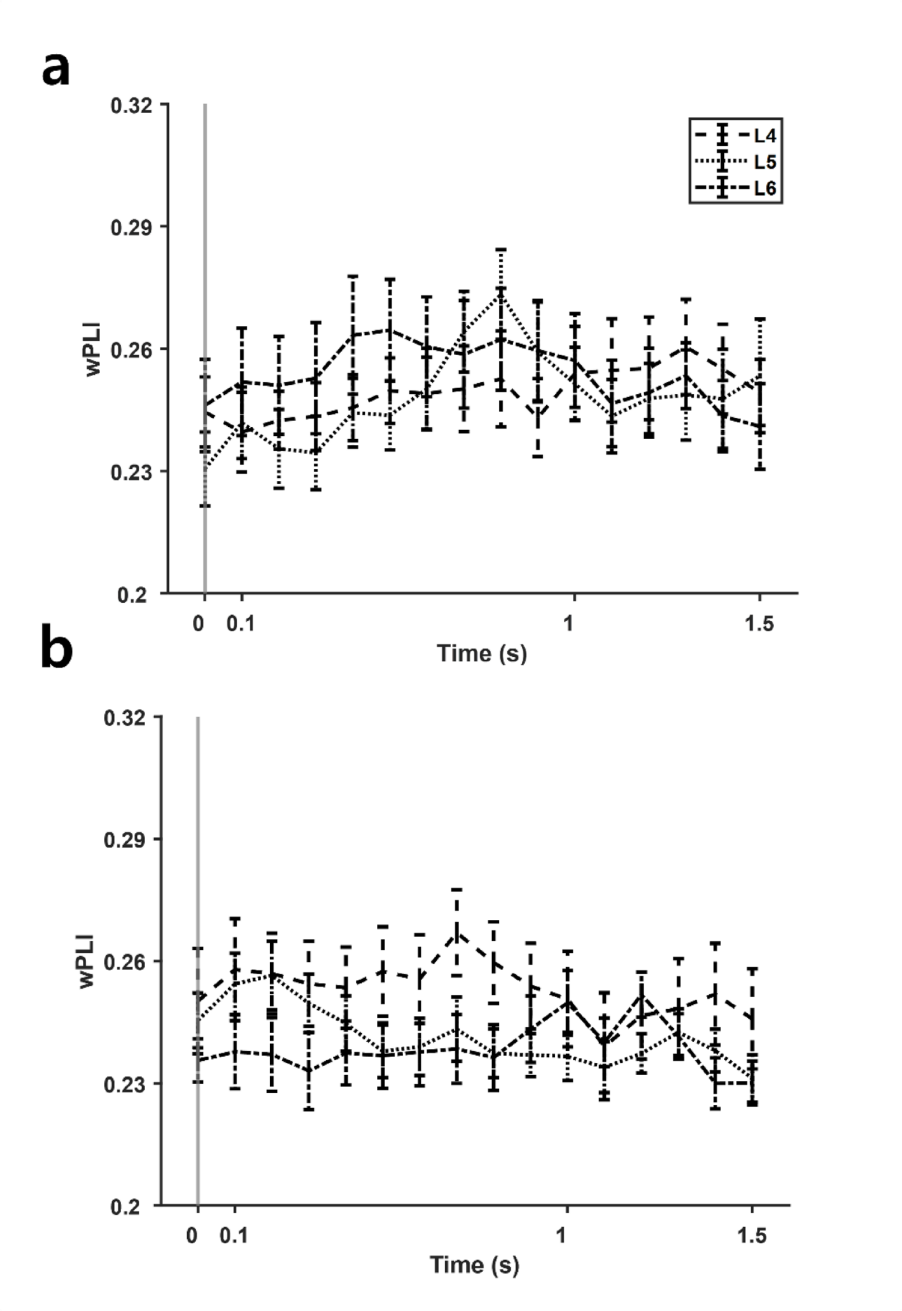
The weighted phase lag index (wPLI) values of the P1-P2 electrode pair during task for loads 4, 5, and 6 (L4, L5, and L6, respectively), with grey line at time point 0 indicating onset of sample stimulus, computed for **a** high gamma band and **b** low gamma band. No significant difference was observed for all segments. Error bars denote standard errors for each segment

To test whether this high gamma band connectivity was directly related to working memory load, we separated the trials by load and observed difference between the wPLI values respective to the loads. If the high gamma connectivity observed were product of item storage, the wPLI during retention period is expected to show a load-dependent trend. Since significant task modulated gamma activity was observed for electrode pairs P1-P2, we primarily report the load-dependent effects for the electrode pairs (Fig. 4). One-way analysis of variance (ANOVA) for within factor load did not show any significant difference, for both low gamma band (P1–P2: *F*_*2, 36*_ < 1.59, *p* > 0.21) and high gamma band (P1–P2: *F*_*2, 36*_ < 0.42, *p* > 0.66). Furthermore, we did not find any consistent load-dependent trend (i.e., higher wPLI for higher load during retention) of wPLI. Combined, the increased functional connectivity between the bilateral parietal regions reflects strengthened phase-lagged synchronization during the retention phase of VWM but not affected by the number of items stored. No other symmetric electrode pairs showed significant interactions either (O1-O2: *F*_*2, 36*_ > 1.25, *p* > 0.29; P5–P6: *F*_*2, 36*_ < 2.17, *p* > 0.12; PO7–PO8: *F*_*2, 36*_ > 2.06, *p* > 0.13 for low gamma; O1-O2: *F*_*2, 36*_ < 0.66, *p* > 0.52; P5–P6: *F*_*2, 36*_ < 1.45, *p* > 0.24; PO7–PO8: *F*_*2, 36*_ < 1.14, *p* > 0.32 for high gamma).

**Fig. 5:**
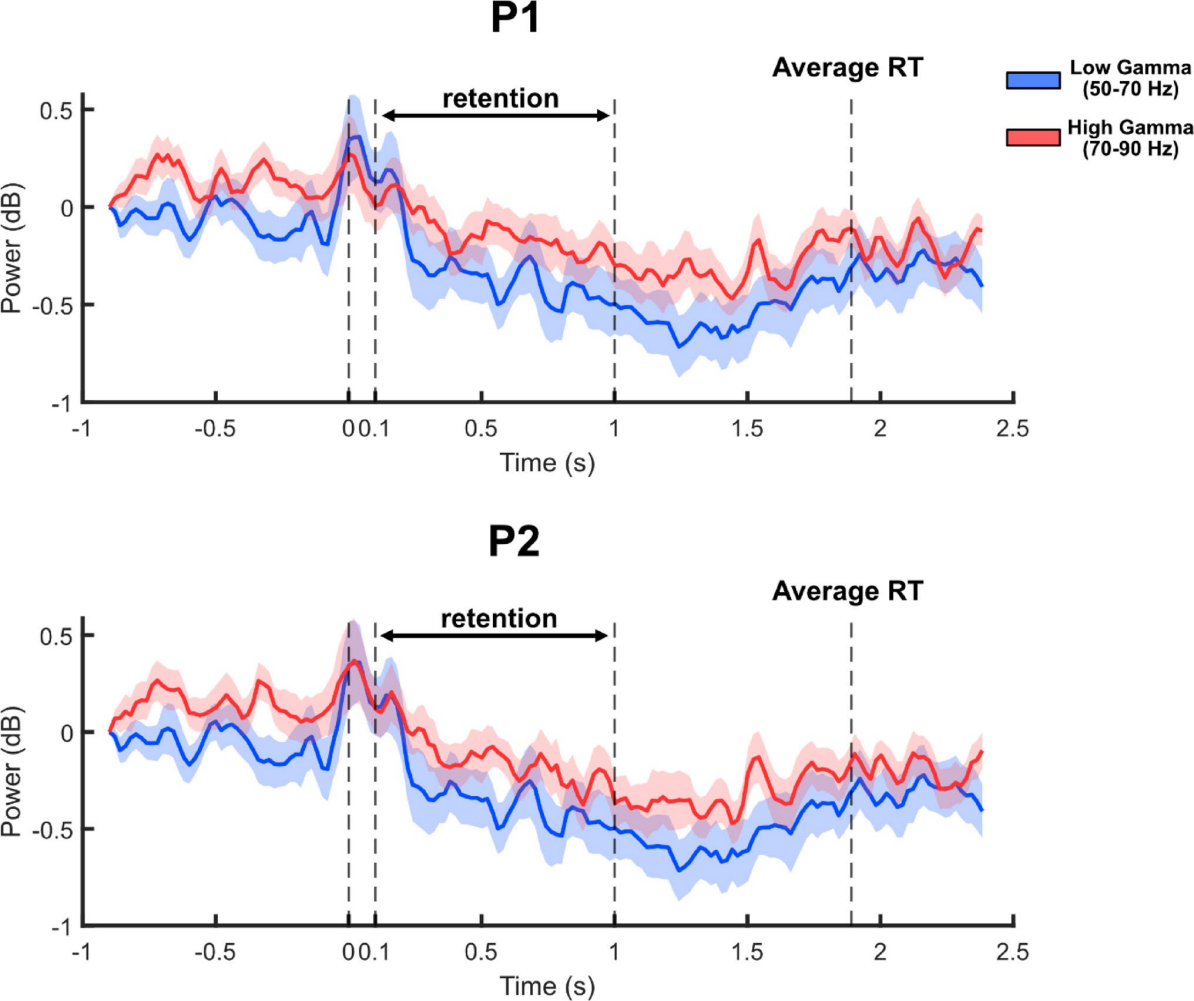
Average power of high and low gamma activity across trials, at electrodes positioned over P1 and P2. Timepoint 0 indicates stimulus onset. Both electrodes show ERS in response to visual stimulus presentation (0 s), while ERD is observed during retention period (0.1–1 s) for both low (50–70 Hz) and high (70–90 Hz) gamma bands. Shaded regions represent the standard error of the mean across participants

Finally, we tested whether this change in gamma connectivity was related to manipulation and processing was related to processing of visual inputs in electrode P1 and P2 (figure 5). If the gamma activity were modulated by information processing, the gamma power would show event related change, especially during retention period. Indeed, the gamma power showed ERD during retention period and ERS after visual stimulus onsets (t = 0 and t = 0.1 seconds. Overall, both low and high gamma showed ERD during retention, suggesting that the gamma rhythms reflects the brain state during information processing and manipulation.

## Discussion

In this study, we observed functional connectivity in the bilateral parietal areas during VWM performance using EEG. We recorded EEG signals from eight electrode sites, four from each left and right hemisphere. P1 and P2 are known to represent the activity from the IPS [20, 21]. Indeed, we found that the wPLI values of high gamma band signals recorded from P1 and P2 increased, only for the high gamma band. While this is not direct evidence of phase delay over two recorded sites, it may indicate that signals from the recorded sites exhibit a systematic non-zero phase relationship. Putatively, 90° at 80 Hz is only approximately 3 ms. However, a reverberate neuronal model suggests that a delay as small as 2 ms is critical for the retention of visual memory [22]. Furthermore, Besserve et al. [23] reported stimulus-modulated phase delays of the high gamma band in the primate visual cortex during performing visual tasks. Taken together, increased functional connectivity observed in the current study and the previous tACS study raises the possibility that the phase shift of the high gamma range in parietal areas, especially during the retention period of VWM, may reflect a mechanism to support processing of visual items during VWM tasks.

Based on these understandings, we observed the correlation between LI of VWM capacity and the wPLI values of high gamma band. We mainly reported high gamma connectivity because high gamma activities are reported to reflect broadband signal arising from populational activity of neurons that can align when cortical regions receive temporally coordinated input [24]. Meanwhile, low gamma activity reflects micro circuit loops which is expected to synchronize when stable rhythmic oscillations are maintained across regions [25]. The latter, however, is unlikely since EEG activities show reduced power, or ERD, to allow for more flexible and information-efficient processing state during retention period [26, 27]. This ERD during retention period is replicated in Fig. 5. In turn, we accounted for this explanation when analyzing behavioral performances by observing interaction between inputs from different visual hemifield, in the form of LI. Combined with the studies reporting that systematic delays in the gamma band entail the direction of the communication between the cortical regions [25, 28, 29], we cautiously propose that such a shift in LI may reflect a behavioral correlate of altered weighting across inputs to bilateral parietal areas, potentially reflecting a compensatory strengthening of previously weaker inputs.

Subsequently, we further explored whether the laterality related connectivity change was related to specific components of working memory. Conventionally, working memory processes are thought to involve two distinct components: item storage and information processing [30]. We first hypothesized that the wPLI values would show load dependent increase if the intraparietal gamma connectivity was related to item storage. Indeed, contralateral bias of parietal sites when performing VWM tasks has been reported, both bilateral parietal areas are considered responsible for the binding of VWM [31–33]. Moreover, the correlation was stronger during the retention period and the start of the retrieval period, when stored information is processed by other regions for manipulation [34]. However, the wPLI values were not significantly different for trials with different loads or for different hemifield trials. The lack of significance for the ‘load’ factor suggests that the phase delay observed over bilateral parietal sites was strictly related to the VWM process itself, not to the level of cognitive demand of the task. For the electrode pair–P1-P2, the wPLI values during the task are separately illustrated for each load in Fig. 4. Meanwhile, information processing is often reflected in ERD/ERS of spectral power density [26, 35]. Time frequency analysis revealed ERS when visual stimulus was presented and ERD during retention period for both high and low gamma bands.

Considering these results, inter-regional activities of the cortex may provide another perspective on behavioral lateralization other than VWM, such as right ear advantage or handedness. Granted, msmp-tACS could serve as a useful approach to explore the relationships between behavioral lateralization, inter-regional phase relationships, and inter-hemispheric phase asymmetries. Clinically, the right ear advantage is known to be significantly decreased in patients with schizophrenia with auditory-verbal hallucinations (AVH) [36–38] because of changes in the gamma band. However, both in-phase and anti-phase gamma tACS of the bilateral auditory cortices failed to modulate the right ear advantage [39]; utilizing msmp-tACS of gamma range may enhance right ear advantage, and in turn may help alleviate AVH symptoms.

Despite the findings, several limitations warrant consideration. First, while gamma activities, especially the high gamma band, are thought to reflect populational firing, the current results lack direct translation from intracranial recordings. Therefore, the frequency ranges and proposed mechanisms are limited to correlational evidence. Second, use of only eight electrodes on the posterior site restricts from interpreting the results as large-scale network level effect. Accordingly, the interpretation of the central findings is limited to synchronization of local activities—which are, in fact, characterized by high gamma activity. Finally, imbalance in gender distribution of our samples limits generalizability. Although there are reports suggesting negligible differences between genders for working memory performance and its correlation to EEG signatures [40, 41], large-scale studies to establish such results are still absent. Note that this limitation is unlikely to affect our core findings, as our analyses rely on within-subject contrasts, which are generally robust to between-group demographic factors, including gender.

## References

[1] Woodman GF, Luck SJ. Visual search is slowed when visuospatial working memory is occupied. Psychon Bull Rev. 2004;11(2):269–74. 15260192 10.3758/bf03196569

[2] Hollingworth A, Richard AM, Luck SJ. Understanding the function of visual short-term memory: transsaccadic memory, object correspondence, and gaze correction. J Exp Psychol Gen. 2008;137(1):163–81. 18248135 10.1037/0096-3445.137.1.163 PMC2784885

[3] Fukuda K, Vogel EK. Individual differences in recovery time from attentional capture. Psychol Sci. 2011;22(3):361–8. 21310945 10.1177/0956797611398493 PMC4494671

[4] Kane MJ, Engle RW. The role of prefrontal cortex in working-memory capacity, executive attention, and general fluid intelligence: an individual-differences perspective. Psychon Bull Rev. 2002;9(4):637–71. 12613671 10.3758/bf03196323

[5] Silvanto J, Cattaneo Z. Transcranial magnetic stimulation reveals the content of visual short-term memory in the visual cortex. Neuroimage. 2010;50(4):1683–9. 20079448 10.1016/j.neuroimage.2010.01.021 PMC3221046

[6] Zaehle T, Sandmann P, Thorne JD, Jäncke L, Herrmann CS. Transcranial direct current stimulation of the prefrontal cortex modulates working memory performance: combined behavioural and electrophysiological evidence. BMC Neurosci. 2011;12(1):2. 21211016 10.1186/1471-2202-12-2 PMC3024225

[7] Klingberg T. Development of a superior frontal–intraparietal network for visuo-spatial working memory. Neuropsychologia. 2006;44(11):2171–7. 16405923 10.1016/j.neuropsychologia.2005.11.019

[8] Tseng P, Iu KC, Juan CH. The critical role of phase difference in theta oscillation between bilateral parietal cortices for visuospatial working memory. Sci Rep. 2018;8(1):349. 29321584 10.1038/s41598-017-18449-w PMC5762658

[9] Jaušovec N, Jaušovec K, Pahor A. The influence of theta transcranial alternating current stimulation (tACS) on working memory storage and processing functions. Acta Psychol. 2014;146:1–6. 10.1016/j.actpsy.2013.11.011 24361739

[10] Wolinski N, Cooper NR, Sauseng P, Romei V. The speed of parietal theta frequency drives visuospatial working memory capacity. PLoS Biol. 2018;16(3):e2005348. 29538384 10.1371/journal.pbio.2005348 PMC5868840

[11] Pahor A, Jaušovec N. The effects of theta and gamma tACS on working memory and electrophysiology. Front Hum Neurosci. 2018;10(11):651. 10.3389/fnhum.2017.00651 PMC5767723 29375347

[12] Kleinert ML, Szymanski C, Müller V. Frequency-unspecific effects of θ-tACS related to a visuospatial working memory task. Front Hum Neurosci. 2017;12(11):367. 10.3389/fnhum.2017.00367 PMC5506205 28747881

[13] Neuling T, Ruhnau P, Weisz N, Herrmann CS, Demarchi G. Faith and oscillations recovered: on analyzing EEG/MEG signals during tACS. Neuroimage. 2017;147:960–3. 27888060 10.1016/j.neuroimage.2016.11.022

[14] Chander BS, Witkowski M, Braun C, Robinson SE, Born J, Cohen LG, et al. tACS phase locking of frontal midline theta oscillations disrupts working memory performance. Front Cell Neurosci. 2016. 10.3389/fncel.2016.00120/abstract. 10.3389/fncel.2016.00120 PMC4858529 27199669

[15] Polanía R, Moisa M, Opitz A, Grueschow M, Ruff CC. The precision of value-based choices depends causally on fronto-parietal phase coupling. Nat Commun. 2015;6(1):8090. 26290482 10.1038/ncomms9090 PMC4560799

[16] Preisig BC, Riecke L, Sjerps MJ, Kösem A, Kop BR, Bramson B, et al. Selective modulation of interhemispheric connectivity by transcranial alternating current stimulation influences binaural integration. Proc Natl Acad Sci USA. 2021;118(7):e2015488118. 33568530 10.1073/pnas.2015488118 PMC7896308

[17] Park J, Lee S, Park S, Lee C, Kim S, Im CH. Transcranial alternating current stimulation over multiple brain areas with non-zero phase delays other than 180 degrees modulates visuospatial working memory performance. Sci Rep. 2023;13(1):12710. 37543713 10.1038/s41598-023-39960-3 PMC10404219

[18] Vogel EK, Machizawa MG. Neural activity predicts individual differences in visual working memory capacity. Nature. 2004;428(6984):748–51. 15085132 10.1038/nature02447

[19] Vinck M, Oostenveld R, van Wingerden M, Battaglia F, Pennartz CMA. An improved index of phase-synchronization for electrophysiological data in the presence of volume-conduction, noise and sample-size bias. Neuroimage. 2011;55(4):1548–65. 21276857 10.1016/j.neuroimage.2011.01.055

[20] Emrich SM, Johnson JS, Sutterer DW, Postle BR. Comparing the effects of 10-Hz repetitive TMS on tasks of visual STM and attention. J Cogn Neurosci. 2017;29(2):286–97. 27626224 10.1162/jocn_a_01043 PMC5199610

[21] Capotosto P, Spadone S, Tosoni A, Sestieri C, Romani GL, Della Penna S, et al. Dynamics of EEG rhythms support distinct visual selection mechanisms in parietal cortex: a simultaneous transcranial magnetic stimulation and EEG study. J Neurosci. 2015;35(2):721–30. 25589765 10.1523/JNEUROSCI.2066-14.2015 PMC4293418

[22] Raffone A, Wolters G. A cortical mechanism for binding in visual working memory. J Cogn Neurosci. 2001;13(6):766–85. 11564321 10.1162/08989290152541430

[23] Besserve M, Lowe SC, Logothetis NK, Schölkopf B, Panzeri S. Shifts of gamma phase across primary visual cortical sites reflect dynamic stimulus-modulated information transfer. PLoS Biol. 2015;13(9):e1002257. 26394205 10.1371/journal.pbio.1002257 PMC4579086

[24] Different Origins of Gamma Rhythm and High-Gamma Activity in Macaque Visual Cortex. PLOS Biol. Available from: 10.1371/journal.pbio.1000610 10.1371/journal.pbio.1000610 PMC3075230 21532743

[25] Bastos AM, Vezoli J, Fries P. Communication through coherence with inter-areal delays. Curr Opin Neurobiol. 2015;31(1):173–80. 25460074 10.1016/j.conb.2014.11.001

[26] Pfurtscheller G, Lopes da Silva FH. Event-related EEG/MEG synchronization and desynchronization: basic principles. Clin Neurophysiol. 1999;110(11):1842–57. 10576479 10.1016/s1388-2457(99)00141-8

[27] Hanslmayr S, Staudigl T, Fellner MC. Oscillatory power decreases and long-term memory: the information via desynchronization hypothesis. Front Hum Neurosci. 2012. 10.3389/fnhum.2012.00074/full. 10.3389/fnhum.2012.00074 PMC3322486 22514527

[28] Grothe I, Neitzel SD, Mandon S, Kreiter AK. Switching neuronal inputs by differential modulations of gamma-band phase-coherence. J Neurosci. 2012;32(46):16172–80. 23152601 10.1523/JNEUROSCI.0890-12.2012 PMC6794021

[29] Gregoriou GG, Gotts SJ, Zhou H, Desimone R. High-frequency, long-range coupling between prefrontal and visual cortex during attention. Science. 2009;324(5931):1207–10. 19478185 10.1126/science.1171402 PMC2849291

[30] Baddeley A. Working memory: theories, models, and controversies. Ann Rev Psychol. 2012;63:1–29. 21961947 10.1146/annurev-psych-120710-100422

[31] Feng J, Pratt J, Spence I. Attention and visuospatial working memory share the same processing resources. Front Psychol. 2012. 10.3389/fpsyg.2012.00103/abstract. 10.3389/fpsyg.2012.00103 PMC3328810 22529826

[32] McCollough AW, Machizawa MG, Vogel EK. Electrophysiological measures of maintaining representations in visual working memory. Cortex. 2007;43(1):77–94. 17334209 10.1016/s0010-9452(08)70447-7

[33] Killebrew K, Mruczek R, Berryhill ME. Intraparietal regions play a material general role in working memory: evidence supporting an internal attentional role. Neuropsychologia. 2015;73:12–24. 25940098 10.1016/j.neuropsychologia.2015.04.032 PMC4468015

[34] Ruchkin DS, Grafman J, Cameron K, Berndt RS. Working memory retention systems: a state of activated long-term memory. Behav Brain Sci. 2003;26(6):709–28. 15377128 10.1017/s0140525x03000165

[35] Klimesch W, Doppelmayr M, Pachinger T, Russegger H. Event-related desynchronization in the alpha band and the processing of semantic information. Cogn Brain Res. 1997;6(2):83–94. 10.1016/s0926-6410(97)00018-9 9450602

[36] Green M, Hugdahl K, Mitchell S. Dichotic listening during auditory hallucinations in patients with schizophrenia. AJP. 1994;151(3):357–62. 10.1176/ajp.151.3.357 8109643

[37] Hugdahl K, L⊘berg EM, J⊘rgensen HA, Lundervold A, Lund A, Green MF, et al. Left hemisphere lateralisation of auditory hallucinations in schizophrenia: a dichotic listening study. Cogn Neuropsychiatry. 2008;13(2):166–79. 18302028 10.1080/13546800801906808

[38] Steinmann S, Leicht G, Andreou C, Polomac N, Mulert C. Auditory verbal hallucinations related to altered long-range synchrony of gamma-band oscillations. Sci Rep. 2017;7(1):8401. 28827744 10.1038/s41598-017-09253-7 PMC5566404

[39] Meier J, Nolte G, Schneider TR, Engel AK, Leicht G, Mulert C. Intrinsic 40Hz-phase asymmetries predict tACS effects during conscious auditory perception. PLoS ONE. 2019;14(4):e0213996. 30943251 10.1371/journal.pone.0213996 PMC6447177

[40] Mental Rotational Ability Is Correlated with Spatial but Not Verbal Working Memory Performance and P300 Amplitude in Males. PLOS One. 2025. Available from: https://journals.plos.org/plosone/article?id=10.1371%2Fjournal.pone.0057390&utm_source=chatgpt.com. 10.1371/journal.pone.0057390 PMC3577737 23437381

[41] Gevins A, Smith ME. Neurophysiological measures of working memory and individual differences in cognitive ability and cognitive style. Cereb Cortex. 2000;10(9):829–39. 10982744 10.1093/cercor/10.9.829

